# The relationship between private import of alcoholic beverages and domestic sales of alcoholic beverages: analyses of Swedish time-series data

**DOI:** 10.1093/eurpub/ckaf139

**Published:** 2025-08-07

**Authors:** Håkan Leifman, Thor Norström

**Affiliations:** Department of Clinical Neuroscience, Karolinska Institutet, Stockholm, Sweden; Swedish Institute for Social Research, Stockholm University, Stockholm, Sweden

## Abstract

Private imports of alcoholic beverages—often referred to as cross-border trade—have long raised concerns in Sweden and other Nordic countries due to their potential impact on national alcohol policies and public health. A key concern is that this trade increases total alcohol consumption and related harms. This study examines the relationship between private alcohol imports and domestic sales in Sweden, with a focus on how cross-border trade influences total alcohol consumption. Using regional and beverage-specific time series data from 2002 to 2023, we applied SARIMA modelling to self-reported import estimates from the Monitor project to assess substitution effects on domestic alcohol sales. Private imports significantly displace domestic alcohol purchases, particularly in southern Sweden across all beverages, though the substitution is partial. For Sweden as a whole, analyses indicate that a 1-litre increase in imports is associated with a 0.456-litre decrease in domestic sales. In central Sweden, only spirits imports show a significant effect (1-litre increase in spirits imports yields a 0.175-litre decrease in domestic sprits sales), while no significant associations are observed in the north. The findings suggest that cross-border trade contributes to higher overall alcohol consumption, especially in border regions. Regional variation underscores the need for differentiated alcohol policy responses. Private imports undermine the effectiveness of domestic alcohol control measures. However, since domestic sales do not influence import levels, policy efforts such as increased excise taxation may reduce total consumption without triggering substantial compensatory imports.

## Introduction

The impact of large-scale private imports of alcoholic beverages on national alcohol policies and public health has long been debated in Sweden and other Nordic countries. A key concern is that this trade—often called cross-border trade—increases total alcohol consumption and related harms. Yet, the relationship between cross-border trade and domestic alcohol sales remains poorly understood. This paper addresses this gap by analysing Swedish time-series data from Q1 2002 to Q4 2023.

Sweden has a long history of restrictive alcohol policies aiming to curtail alcohol consumption and alcohol-related harms. However, cross-border trade threatens to undermine these efforts by providing access to cheaper alcohol, thereby potentially increasing overall consumption and related harms. A recent overview study on the cross-border trade of alcoholic beverages within the European Union identified three main concerns: public health, fraudulent behaviour such as smuggling, and economic distortion [1]. These concerns are largely driven by the following factors: (a) the high price sensitivity of alcoholic beverages [2, 3], (b) substantial differences in excise duties and prices between EU countries [1], and (c) the legal possibility within the EU to transport unlimited quantities of alcohol for personal use across borders without paying additional excise duties in the destination country [1].

From a public health perspective, extensive inflow of alcohol through cross-border trade may lead to an increase in total alcohol consumption and associated harms. This can occur through two main processes: first, by directly increasing accessibility to cheaper alcohol, and second, by discouraging the use of excise duties as a public health tool for fear of losing purchases to neighbouring states. Empirical data—and most, though not all, studies—from northern European countries with high levels of private import suggest that such inflow, through one or both of the mechanisms, has contributed to both higher alcohol consumption and weakened alcohol policy measures [4–9]. Reflecting the latter mechanism, Denmark, Estonia, Finland, and Sweden have over the past decades reduced alcohol taxes—either through explicit cuts (e.g. Sweden in 1997; Denmark in 2003 and 2019; Finland in 2004) or by keeping nominal rates unchanged despite inflation [10].

These decisions were often based on the assumption that domestic sales respond directly to changes in private imports. However, empirical evidence on the nature and strength of this relationship remains limited.

In Sweden, trends over the past 20 years have shown a negative correlation between domestic sales and cross-border sales. However, trends alone cannot be used as evidence of causation. The COVID-19 pandemic provided a unique natural experiment: with travel restrictions in place, cross-border trade sharply declined, domestic retail sales increased, and total per capita alcohol consumption decreased in countries previously characterized by significant alcohol inflows such as Sweden [10]. However, the exceptional nature of pandemic conditions limits the generalizability of these observations.

Other studies have examined the relationship between cross-border shopping and distance from the border, using varying methods and data types [11–14]. Leal *et al.*’s meta-analysis [13] and Norström’s study [14] found that cross-border shopping decreases with greater distance from the border. Asplund *et al.* [11] analysed how Swedish alcohol sales respond to foreign prices and proximity to Denmark and Germany, estimating price elasticities of about –0.3 in border areas and –0.2 further inland. Crawford and Tanner [12] showed that demand for wine in southeast England became more price elastic after the EU Single Market was introduced in 1993. Proximity to continental Europe amplified substitution effects, especially near ports and the Channel Tunnel. However, cross-border shopping only partially replaced domestic purchases, particularly for wine, suggesting the effect is not zero-sum.

However, none of the existing studies have access data encompassing both cross-border trade and domestic sales. This highlights the need for additional research, particularly time series analyses that explore the direct relationship between changes in cross-border trade and domestic sales.

High-quality data on both private imports and domestic sales are available for Sweden [15], but no study to date has exploited this resource to directly analyse the relationship between the two.

### Aim of the study

This study estimates the relationship between private imports and domestic sales of alcoholic beverages in Sweden. While a positive relationship is possible—reflecting changes in overall alcohol demand—it is more likely that the two act as substitutes due to consumers’ budget constraints, a view supported by previous empirical evidence [16]. A key question, therefore, is the direction of this relationship: do changes in private imports influence domestic sales, or is the reverse true?

One possible mechanism is that when individuals bring home alcohol from abroad, their subsequent demand for domestic alcohol may decline due to limited storage space, budgetary limits, or simply a reduced need to purchase more. Conversely, an increase in domestic alcohol purchases—for example, in connection with a celebration—may not necessarily deter future private imports, as these purchases often occur independently of one another. This asymmetry would suggest that changes in private imports could have a stronger effect on domestic sales than the other way around.

However, the relationship may vary across regions. In border areas, price-conscious consumers have easier access to alcohol from neighbouring countries, making private imports a routine rather than occasional activity. In these cases, fluctuations in domestic sales—due to price changes, availability, or policy interventions—may more strongly influence private imports than in other regions. This points to a potentially bidirectional relationship and underscores the need to consider regional variation when analysing these dynamics. Both mechanisms may thus be present simultaneously, especially in border regions, raising the question of which is dominant.

The strength of these mechanisms may also vary by beverage type. Spirits are more commonly imported during regular trips abroad—typically by air travel—and thus align with general travel trends. Beer is primarily brought home through border trade, especially from northern Germany, while wine is imported via both channels [17, 18]. These differences indicate that import patterns vary by beverage type and should be considered in the analysis of private imports and domestic sales.

## Methods

### Data

We used two sources of alcohol data:

1. Domestic alcohol sales were measured by the sales of Systembolaget, Sweden’s government alcohol retail monopoly for beverages over 2.25% (except beer up to 3.5% ABV sold in grocery stores). As of 2023, Systembolaget operated approximately 450 outlets, with a stable density of ∼0.52–0.53 stores per 10 000 inhabitants aged 15+.
2. Private imports were estimated using data from the Monitor project, a monthly survey initiated in 2000 that collects self-reported alcohol consumption and unrecorded purchases. Respondents (aged 17–84) are randomly selected, and ∼1500 interviews are completed monthly (∼18 000/year), though response rates declined from ∼60% to 28% by 2023 [15]. Since 2020, respondents have been able to reply via digital forms or telephone, whereas previously responses were collected by telephone only. This change in methodology had a slight impact which, however, has been accounted for in the estimates of imports [15]. Alcohol import volumes were then estimated based on self-reports of travel abroad during the past 30 days, including the number of trips, whether alcohol was brought back, and details on beverage types (beer, wine, spirits, cider/alcopops), volumes, and packaging sizes. For shared imports, reported volumes were proportionally distributed among the group to prevent double counting and overestimation [15].

All purchase data (Systembolaget and import) were converted to litres of 100% alcohol per capita (aged 15+), with indicators for total volume and by beverage type. The dataset covers Q1 2002 to Q4 2023. Given known geographic variation—particularly stronger cross-border effects in southern regions—we analysed data at both national and regional levels:

1. Southern Sweden (counties): Skåne, Blekinge, Halland, Östergötland, Jönköping, Kronoberg, Kalmar, Västra Götaland.
2. Mid-Sweden (counties): Stockholm, Uppsala, Södermanland, Värmland, Örebro, Västmanland.
3. Northern Sweden (counties): Gotland, Dalarna, Gävleborg, Västernorrland, Jämtland, Västerbotten, Norrbotten.

We included the following control variables:

1. Beverage-specific excise duty rates expressed in Swedish krona per litre pure (100%) alcohol for beer and spirits and in litres for the commonly sold wines (those >8%–15% by volume which, accounts for more than 96% all wine sold at Systembolaget).
2. COVID-19 which was a dummy variable taking the value 1 the years 2020 and 2021, and 0 otherwise.

### Statistical analyses

*Assessing the lag structure.* The initial analyses aimed to determine the directionality and lag structure of the relationship between domestic sales and imports. We began by calculating cross-correlations for various lags of the input series (imports). These cross-correlations, typically denoted as CCF(k), represent the correlation at different lag intervals, where k indicates the lag. Specifically, CCF(0) represents the instantaneous correlation between the two series, while CCF(1) denotes the correlation when the input series is lagged by one observation, which can be extended to higher lag values. Significant cross-correlations at higher lags indicate the presence of a lag structure. A negative lag 0 correlation indicates that import and sales are negatively related, but the direction of the association cannot be determined. A statistically significant negative coefficient at lag 1 suggests that an increase in import a particular quarter tends to decrease domestic sales during the following quarter.

To avoid spurious correlations driven by autocorrelation within the series, all cross-correlations were computed on pre-whitened data. This was performed by fitting univariate SARIMA models (see below) to the various alcohol indicators and extracting the residuals from these models. The Box-Ljung Q statistics [19] was used to confirm that the residuals represented white noise.

The results, however, were inconclusive. While the cross-correlations at lag 0 were negative, as expected, and generally statistically significant, the cross-correlations at higher lags were insignificant and showed no systematic pattern. In conclusion, these findings did not reveal a clear lag structure or directionality of the association.

Given these limitations, we adopted an alternative modelling approach. Assuming a potential causal pathway from imports to domestic sales, we constructed a lag-weighted import series, capturing the idea that import volumes may reduce domestic purchases not only in the same quarter but also in subsequent ones, with gradually declining influence. To test this, we developed a weighted input series using past and present observations, applying geometrically declining lag weights. The weighted series (*IW*) was constructed as follows:


(1) $${IW}_{t}=I_{t}+{\lambda *I}_{t-1}+\lambda^{2}*I_{t-2}+\lambda^{3}*I_{t-3}$$

where *I* is imports. As shown, there was a truncation at lag 3 (the lag weights were rescaled to sum to unity). The optimal value of the lag parameter (λ) was determined through a search procedure, with λ fixed at values between 0.5 and 0.9.

To test the alternative causal direction—that increased domestic sales would lead to reduced imports—we constructed a weighted input series corresponding to the one described above but based on domestic sales data.

In the next step, we estimated a series of SARIMA models (by region, beverage, and overall sales) using alternating input and output series. We employed a linear model specification, as the resulting estimates are the easiest to interpret and most effectively address the question of how a 1-litre change in imports impacts domestic sales (and how a 1-litre change in domestic sales impacts imports).

The relationship between domestic sales and imports was analysed using SARIMA-models (seasonal autoregressive integrated moving average model) [20]. Non-stationarity in the form of time trends was removed by regular or seasonal differencing. The noise (error) term, which includes explanatory variables not considered in the model, was modelled to account for temporal structure via autoregressive and moving average parameters, both regular and seasonal. A SARIMA-model is denoted (p, d, q) (P, D, Q, M), where the first set of parameters refers to the non-seasonal (regular) components, and the second set to seasonal components. The order of the autoregressive parameter in the model’s non-seasonal part is indicated by p, while d indicates the order of regular differencing, and q is the order of the moving-average parameter. The symbols in the second bracket have the corresponding seasonal significance, while M is the number of periods per season. Model adequacy was assessed using the Box–Ljung Q test to ensure that residuals did not differ significantly from white noise [19].

To sum up, we estimated two sets of models by region and beverage:


(2) $$D_{t}=b_{1}{*IW}_{t}+N_{t}$$

where *D* denotes domestic sales, *IW* represents imports (weighted), and *N* is the noise term. The parameter to be estimated ($b_{1}$) expresses the impact of a 1-litre increase in imports on domestic sales. The second model was:


(3) $$I_{t}=b_{2}*{DW}_{t}+N_{t}$$

where *I* denote imports, *DW* represents domestic sales (weighted), and *N* is the noise term. The parameter to be estimated ($b_{2}$) expresses the impact of a 1-litre increase in domestic sales on imports.

## Results

**Figure 1.**
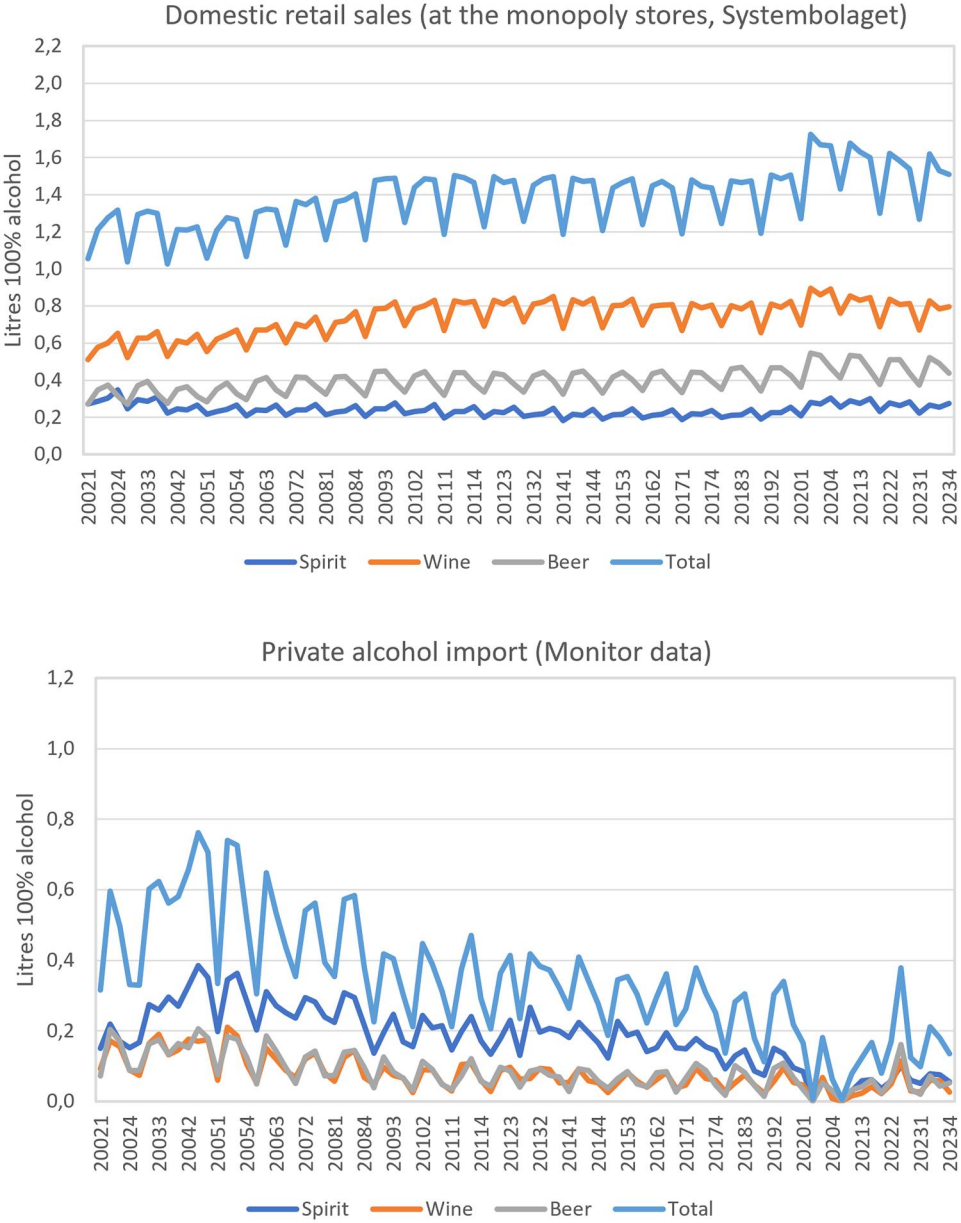
Trends in domestic retail sales and private import of alcohol (per alcoholic beverage and total), per inhabitants 15+, quarterly data from 2002 to 2023.

Figure 1 illustrates the trends in domestic sales at Systembolaget and private alcohol imports, both by beverage type and overall, for Sweden as a whole. Private alcohol imports rose sharply in the early years (2002–2004), coinciding with the expansion of traveller allowances (quotas on travellers’ imports) and, from 2004, the removal of all limits on personal imports. Since 2005, private alcohol imports have gradually declined.

Spirits account for the highest import volumes, while wine is the lowest. At Systembolaget, the pattern is reversed, with wine recording the highest sales volumes and spirits the lowest. These trends are consistent across all three regions, though at different levels—imports are highest in southern Sweden and lowest in the north, whereas Systembolaget’s sales show the opposite pattern. Nevertheless, sales have increased across all regions.

As previously noted, cross-correlations at lag 0 indicate a negative contemporaneous relationship between domestic sales and imports, but they do not establish causal direction, which is examined below.

Table 1 presents the results of the time series (SARIMA) analyses. A lag weight of 0.8 yielded the best model fit with the narrowest confidence intervals around the estimates. The first segment of Table 1 shows that a 1-litre increase in private beer imports in southern Sweden corresponds to a 0.251-litre decrease in domestic beer sales. (Conversely, a 1-litre reduction in imports was associated with a 0.251-litre increase in sales.) In contrast, changes in domestic beer sales had no statistically significant effect on private imports.

**Table 1.** Estimated relationships between private import and domestic sales of alcoholic beverages

						
Output	Input	EST	SE	p	Q	p(Q)	Model
**Southern Sweden**							
BeerSB	BeerIMP	−0.251	0.059	<0.001	7.133	0.129	(2,1,0) (2,0,0,4)
	COVID-19	0.034	0.005	<0.001			
	Tax	−0.069	0.070	0.325			
	Euro	0.006	0.004	0.117			
BeerIMP	BeerSB	−0.674	0.630	0.285	3.264	0.515	(2,1,0) (2,0,0,4)
	COVID-19	−0.069	0.026	0.009			
	Tax	0.139	0.251	0.579			
	Euro	−0.020	0.017	0.233			
WineSB	WineIMP	−0.371	0.121	0.002	4.314	0.365	(0,1,1) (2,0,0,4)
	COVID-19	0.048	0.008	<0.001			
	Tax	−0.318	0.124	0.010			
	Euro	0.011	0.005	0.026			
WineIMP	WineSB	−0.367	0.400	0.359	1.403	0.844	(2,1,0) (2,0,0,4)
	COVID-19	−0.041	0.031	0.186			
	Tax	−0.016	0.572	0.977			
	Euro	−0.018	0.021	0.386			
SpiritsSB	SpiritsIMP	−0.197	0.029	<0.001	2.894	0.576	(2,1,0) (2,0,0,4)
	COVID-19	0.021	0.003	<0.001			
	Tax	−0.267	0.392	0.496			
	Euro	−0.005	0.003	0.176			
SpiritsIMP	SpiritsSB	−1.782	1.514	0.239	2.034	0.730	(2,1,0) (2,0,0,4)
	COVID-19	−0.034	0.056	0.546			
	Tax	1.324	2.871	0.645			
	Euro	−0.061	0.019	0.001			
TotalSB	TotalIMP	−0.323	0.057	<0.001	5.057	0.282	(2,1,0) (2,0,0,4)
	COVID-19	0.090	0.014	<0.001			
	Tax	−0.457	0.311	0.142			
	Euro	0.010	0.011	0.344			
TotalIMP	TotalSB	−0.565	0.516	0.273	2.626	0.622	(2,1,0) (3,0,0,4)
	COVID-19	−0.168	0.072	0.019			
	Tax	0.989	1.106	0.371			
	Euro	−0.086	0.057	0.134			
**Mid-Sweden**							
BeerSB	BeerIMP	−0.060	0.164	0.715	3.162	0.531	(0,1,1) (2,0,0,4)
	COVID-19	0.028	0.006	<0.001			
	Tax	−0.089	0.073	0.225			
	Euro	0.004	0.004	0.317			
BeerIMP	BeerSB	0.038	0.372	0.919	6.241	0.182	2,1,0) (2,0,0,4)
	COVID-19	−0.027	0.018	0.130			
	Tax	−0.058	0.086	0.497			
	Euro	0.003	0.009	0.773			
WineSB	WineIMP	−0.108	0.235	0.645	2.492	0.646	(0,1,1) (2,0,0,4)
	COVID-19	0.054	0.009	<0.001			
	Tax	−0.186	0.137	0.173			
	Euro	0.003	0.005	0.565			
WineIMP	WineSB	−0.443	0.287	0.123	7.858	0.097	(2,1,0) (2,0,0,4)
	COVID-19	−0.001	0.013	0.958			
	Tax	−0.245	0.160	0.126			
	Euro	−0.009	0.016	0.583			
SpiritsSB	SpiritsIMP	−0.175	0.047	<0.001	2.536	0.638	(2,1,0) (2,0,0,4)
	COVID-19	0.016	0.004	<0.001			
	Tax	−0.623	0.579	0.282			
	Euro	−0.003	0.004	0.489			
SpiritsIMP	SpiritsSB	−1.196	0.916	0.192	8.300	0.081	(2,1,0) (2,0,0,4)
	COVID-19	−0.022	0.031	0.467			
	Tax	−0.576	2.130	0.787			
	Euro	−0.028	0.016	0.078			
TotalSB	TotalIMP	−0.306	0.139	0.026	3.2561	0.5159	(2,1,0) (2,0,0,4)
	COVID-19	0.066	0.022	0.003			
	Tax	−0.662	0.656	0.313			
	Euro	0.007	0.012	0.527			
TotalIMP	TotalSB	−0.392	0.380	0.302	6.731	0.151	(2,1,0) (2,0,0,4)
	COVID-19	−0.054	0.045	0.230			
	Tax	−0.506	0.542	0.351			
	Euro	−0.036	0.037	0.334			
**Northern Sweden**							
BeerSB	BeerIMP	0.147	0.145	0.311	7.967	0.093	(2,1,0) (2,0,0,4)
	COVID-19	−0.001	0.011	0.902			
	Tax	−0.121	0.084	0.151			
	Euro	0.006	0.005	0.276			
BeerIMP	BeerSB	0.604	0.483	0.212	1.122	0.891	(3,1,0) (2,0,0,4)
	COVID-19	−0.023	0.034	0.494			
	Tax	−0.043	0.191	0.821			
	Euro	−0.010	0.016	0.543			
WineSB	WineIMP	−0.044	0.232	0.849	5.001	0.287	(0,1,1) (3,0,0,4)
	COVID-19	0.005	0.012	0.663			
	Tax	−0.271	0.119	0.023			
	Euro	0.004	0.004	0.356			
WineIMP	WineSB	0.368	0.488	0.450	6.979	0.137	(2,1,0) (2,0,0,4)
	COVID-19	−0.008	0.073	0.914			
	Tax	0.126	0.435	0.771			
	Euro	0.005	0.023	0.833			
SpiritsSB	SpiritsIMP	−0.069	0.071	0.334	7.896	0.096	(2,1,0) (2,0,0,4)
	COVID-19	0.012	0.006	0.036			
	Tax	−0.884	0.573	0.123			
	Euro	0.001	0.004	0.745			
SpiritsIMP	SpiritsSB	−0.832	0.656	0.205	6.074	0.194	(2,1,0) (2,0,0,4)
	COVID-19	−0.027	0.035	0.443			
	Tax	1.276	1.893	0.500			
	Euro	0.005	0.018	0.791			
TotalSB	TotalIMP	−0.082	0.180	0.649	3.354	0.501	(2,1,0) (3,0,0,4)
	COVID-19	−0.008	0.038	0.825			
	Tax	−0.656	0.537	0.221			
	Euro	0.018	0.014	0.212			
TotalIMP	TotalSB	0.208	0.362	0.566	5.270	0.261	(2,1,0) (2,0,0,4)
	COVID-19	−0.057	0.064	0.372			
	Tax	0.187	0.701	0.789			
	Euro	0.012	0.039	0.754			
**All Sweden**							
BeerSB	BeerIMP	−0.337	0.108	0.002	7.443	0.114	(2,1,0) (2,0,0,4)
	COVID-19	0.023	0.007	0.001			
	Tax	−0.091	0.063	0.148			
	Euro	0.006	0.004	0.117			
BeerIMP	BeerSB	−0.174	0.378	0.646	2.031	0.730	(2,1,0) (2,0,0,4)
	COVID-19	−0.055	0.013	<0.001			
	Tax	0.077	0.108	0.477			
	Euro	−0.009	0.010	0.354			
WineSB	WineIMP	−0.643	0.168	<0.001	2.076	0.722	(0,1,1) (3,0,0,4)
	COVID-19	0.034	0.009	<0.001			
	Tax	−0.354	0.121	0.004			
	Euro	0.008	0.004	0.036			
WineIMP	WineSB	−0.337	0.235	0.153	4.404	0.354	(2,1,0) (2,0,0,4)
	COVID-19	−0.023	0.013	0.073			
	Tax	−0.091	0.227	0.689			
	Euro	−0.011	0.013	0.416			
SpiritsSB	SpiritsIMP	−0.270	0.035	<0.001	3.531	0.473	(2,1,0) (2,0,0,4)
	COVID-19	0.016	0.003	<0.001			
	Tax	−0.409	0.361	0.258			
	Euro	−0.003	0.004	0.375			
SpiritsIMP	SpiritsSB	−0.995	0.769	0.196	1.866	0.760	(2,1,0) (3,0,0,4)
	COVID-19	−0.035	0.027	0.197			
	Tax	2.245	2.228	0.314			
	Euro	−0.036	0.018	0.042			
TotalSB	TotalIMP	−0.456	0.087	<0.001	5.466	0.243	(2,1,0) (3,0,0,4)
	COVID-19	0.057	0.020	0.005			
	Tax	−0.617	0.466	0.185			
	Euro	0.012	0.010	0.209			
TotalIMP	TotalSB	−0.597	0.415	0.151	2.446	0.654	(2,1,0) (2,0,0,4)
	COVID-19	−0.100	0.045	0.027			
	Tax	0.063	0.573	0.913			
	Euro	−0.060	0.031	0.055			

Based on SARIMA-analyses of data for the period Q1 2002–Q4 2023. SB denotes domestic sales at Systembolaget, and IMP signifies import.

As also shown in Table 1, private imports had a statistically significant effect on domestic sales for all beverage types and for total imports in both southern Sweden and at the national level. In central Sweden, only spirits imports showed a significant effect, while no significant associations were observed in northern Sweden.

The fact that the estimated negative effects of private imports on domestic sales are less than one (in absolute terms) indicates incomplete substitution. Rather than replacing domestic purchases one-to-one, imports contribute to net increases in total alcohol consumption (ie domestic sales plus private imports) and conversely, a decrease in imports leads to an overall reduction in total consumption.

Domestic sales showed no statistically significant impact on imports for any beverage, in any of the three regions, or at the national level. Additionally, the significant estimates for beer, wine, and spirits did not differ statistically from each other in any region or for Sweden as a whole.

Overall, Table 1 provides strong support for the first mechanism—where private imports influence domestic sales—across a substantial number of models. In contrast, no empirical evidence was found for the alternative mechanism, in which domestic sales would influence private imports.

## Discussion

This study offers new insights into the relationship between private alcohol imports and domestic sales in Sweden, addressing longstanding concerns about the implications of cross-border trade for national alcohol policy. Drawing on detailed time series data from 2002 to 2023, disaggregated by region and beverage category, the analysis supports the hypothesis that private imports partly substitute for domestic purchases. Crucially, however, the substitution is incomplete, implying that cross-border trade contributes to a net increase in total sales (sum of domestic sales and private imports).

These findings align with previous research on alcohol consumption during the COVID-19 pandemic. When intra-European borders were temporarily closed, countries with a substantial cross-border alcohol shopping—such as Sweden—experienced a notable increase in domestic sales. Yet, total alcohol consumption declined, indicating that imported alcohol was not fully replaced by domestic sources [10].

A central contribution of this study is the clarification of the directionality in the relationship between private imports and domestic alcohol sales. Using SARIMA models with lag-weighted import series, the analysis indicates a unidirectional effect: private imports negatively impact domestic sales, but not vice versa. This finding is consistent with economic theory—particularly budget constraint models—which suggest that imported alcohol, often available at lower prices, serves as a substitute for domestic purchases, especially in border regions.

The absence of statistically significant effects of domestic sales on subsequent imports further strengthens this interpretation. Although most coefficients are negative, they do not reach statistical significance, suggesting that purchases from Systembolaget do not influence import behaviour.

### Regional differences and policy relevance

As hypothesized, the displacement effect is strongest in southern Sweden, consistent with its proximity to Denmark and northern Germany—countries with lower alcohol prices and fewer regulatory barriers. Substitution effects were observed across all beverage types in southern Sweden, but only for spirits in central Sweden and for none in northern Sweden. These regional patterns likely reflect both logistical advantages and economic incentives associated with cross-border shopping.

In central Sweden, only spirits imports had a statistically significant effect on domestic sales, likely due to their high alcohol content relative to volume, making them practical to import even on trips not focused on alcohol purchases.

These findings have important policy implications. In regions where imports displace domestic sales, tax-based measures may be less effective, as consumers can substitute with imported alcohol. Moreover, the partial substitution observed indicates a net increase in consumption. Thus, particularly in border regions, national alcohol policies risk being undermined by market forces operating beyond state control.

### Implications for policy and regulation

Several policy implications emerge from the findings. First, domestic alcohol control measures should be complemented by strategies to address cross-border trade, such as strengthened EU coordination on alcohol taxation and stricter enforcement of personal import limits.

Second, the lack of a significant effect of domestic alcohol sales on private imports suggests that measures such as excise tax increases could reduce overall alcohol consumption without triggering a corresponding rise in private imports. Since imports appear unresponsive to changes in domestic sales, reductions in domestic availability—through taxation or other restrictions—may lower total consumption without full substitution via cross-border purchases. This underscores the continued relevance of national policy tools in reducing alcohol-related harm.

### Limitations and future research

Several limitations should be acknowledged. First, despite the use of a long time series and robust statistical methods, the study remains observational. While SARIMA models with lagged variables suggest causal directionality, future research could benefit from quasi-experimental designs to strengthen causal inference.

Second, private import estimates rely on self-reported data from the Monitor project. Although one of Europe’s most comprehensive alcohol surveys, declining response rates raise concerns about selection bias and representativeness. Alcohol consumption is also prone to underreporting due to social desirability and recall bias. While weighting and statistical adjustments are applied, they cannot fully eliminate these limitations. Future studies should aim to triangulate self-reports with alternative data sources—such as tax records or border statistics—to more accurately capture unrecorded alcohol use.

Third, private imports do not include alcohol illegally brought into the country (i.e. smuggling). Although smuggling volumes are estimated within the Monitor project, these figures cannot be disaggregated by quarter and are associated with considerable uncertainty. While a different temporal pattern for smuggling cannot be ruled out, available evidence suggests that annual trends in smuggling generally mirror those of private imports, though total volumes remain substantially lower [15].

Fourth, in northern and central Sweden, several estimates failed to reach statistical significance, despite the generally negative direction of effects. This may reflect low statistical power, small effect sizes, weak signal-to-noise ratios, or inherent limitations of the survey data.

## Conclusion

This study provides evidence that private alcohol imports significantly reduce domestic sales, particularly in southern Sweden. However, the substitution is incomplete, leading to a net increase in total alcohol availability and sales. These findings highlight a fundamental challenge for Swedish alcohol policy: cross-border trade as a driver of alcohol availability.

At the same time, the absence of significant effects of domestic sales on subsequent imports—combined with only partial substitution by private imports—underscores the continued relevance of national measures, such as taxation, to reduce availability and overall alcohol consumption.

Notably, private imports have declined markedly over the past two decades, for reasons that remain unclear. This likely contributes to the observed increase in domestic sales. Travel volume has not decreased (aside from the pandemic), and exchange rate fluctuations alone do not fully explain the trend.

Addressing this challenge will require coordinated efforts at regional, national, and EU levels to strengthen the effectiveness of alcohol control policies.

Conflict of interest: None declared.

## Data Availability

The datasets analysed in this study are available from the corresponding author on reasonable request. Key points Private alcohol imports significantly reduce domestic alcohol sales, particularly in southern Sweden. The substitution effect is incomplete, resulting in higher overall alcohol consumption. National measures like excise taxation remain effective tools for reducing alcohol consumption despite cross-border trade.
